# Phytohormonal Regulation of Biomass Allocation and Morphological and Physiological Traits of Leaves in Response to Environmental Changes in *Polygonum cuspidatum*

**DOI:** 10.3389/fpls.2016.01189

**Published:** 2016-08-09

**Authors:** Daisuke Sugiura, Mikiko Kojima, Hitoshi Sakakibara

**Affiliations:** ^1^Laboratory of Plant Ecology, Department of Biological Sciences, Graduate School of Science, The University of TokyoBunkyo, Japan; ^2^Plant Productivity Systems Research Group, RIKEN Center for Sustainable Resource ScienceYokohama, Japan

**Keywords:** biomass allocation, cytokinins, gibberellins, leaf mass per area, nitrogen, non-structural carbohydrate, photosynthesis

## Abstract

Plants plastically change their morphological and physiological traits in response to environmental changes, which are accompanied by changes in endogenous levels of phytohormones. Although roles of phytohormones in various aspects of plant growth and development were elucidated, their importance in the regulation of biomass allocation was not fully investigated. This study aimed to determine causal relationships among changes in biomass allocation, morphological and physiological traits, and endogenous levels of phytohormones such as gibberellins (GAs) and cytokinins (CKs) in response to environmental changes in *Polygonum cuspidatum*. Seedlings of *P. cuspidatum* were grown under two light intensities, each at three nitrogen availabilities. The seedlings grown in high light intensity and high nitrogen availability (HH) were subjected to three additional treatments: Defoliating half of the leaves (Def), transferral to low nitrogen availability (LowN), or low light intensity (LowL). Biomass allocation at the whole-plant level, morphological and physiological traits of each leaf, and endogenous levels of phytohormones in each leaf and shoot apex were measured. Age-dependent changes in leaf traits were also investigated. After the treatments, endogenous levels of GAs in the shoot apex and leaves significantly increased in Def, decreased in LowN, and did not change in LowL compared with HH seedlings. Among all of the seedlings, the levels of GAs in the shoot apex and leaves were strongly correlated with biomass allocation ratio between leaves and roots. The levels of GAs in the youngest leaves were highest, while the levels of CKs were almost consistent in each leaf. The levels of CKs were positively correlated with leaf nitrogen content in each leaf, whereas the levels of GAs were negatively correlated with the total non-structural carbohydrate content in each leaf. These results support our hypothesis that GAs and CKs are key regulatory factors that control biomass allocation, leaf morphology, and photosynthesis in response to changes in environmental variables in *P*. *cuspidatum*.

## Introduction

Plants plastically adjust biomass allocation between leaves and roots (*L*/*R*, g g^−1^), leaf mass per area (LMA, g m^−2^), and leaf nitrogen content (N_area_, g N m^−2^) to environmental variables such as light intensity and nitrogen availability (Givnish, 1988; Poorter et al., 2012) by modifying the allocation patterns so as to maximize their relative growth rate (RGR, g g^−1^ d^−1^; Hilbert et al., 1991; Osone and Tateno, 2003; Sugiura and Tateno, 2011). It is also known that plants shift their allocation patterns in response to the loss of leaves or roots to restore their original *L*/*R* (Alexander and Maggs, 1971; Poorter et al., 2000). These indicate that plants can recognize the demands for carbon and nitrogen of each organ and regulate the activity of meristematic tissues in response to changes in environmental variables or to loss of organs due to herbivory and disturbances.

Previous studies indicate that phytohormones such as gibberellins (GAs) and cytokinins (CKs) play important roles in the regulation of biomass allocation and leaf morphology. Trans-zeatin types of CKs, known to be long-distance signals of soil nitrogen availability, are mainly synthesized in the roots and translocated to the shoots (Sakakibara, 2003). Plants grown under high nitrogen availability show high *L*/*R* and their endogenous CKs levels are high in both above- and below-ground organs (Beck, 1996; Kiba et al., 2011). Applications of CKs such as benzyl adenine also cause an increase in *L*/*R* (Van der Werf and Nagel, 1996). The increase in *L*/*R* may be due to the promotion of leaf growth and inhibition of lateral root growth by CKs (Werner et al., 2003, 2010; Laplaze et al., 2007; Hachiya et al., 2014).

Gibberellins (GAs) may be involved also in regulating biomass allocation and LMA in addition to their well-known roles in stem and petiole elongation, seed germination, and flowering (Hedden and Thomas, 2012). Genotypes with high levels of endogenous GAs show high *L*/*R* or shoot-to-root ratios and low LMA compared to those with low levels of endogenous GAs in *Zea mays* (Rood et al., 1983), *Populus* sp. (Bate et al., 1988), *Plantago major* (Dijkstra et al., 1990), *Brassica rapa* (Rood et al., 1990), and *Solanum lycopersicum* (Nagel et al., 2001a). Biosynthesis of GAs is regulated by both light quality and quantity via phytochrome (Kamiya and García-Martîez, 1999), and changes in the level of GAs strongly affect the phytochrome-mediated morphogenesis of the leaves and stems (Kurepin et al., 2006, 2012).

Previously, we evaluated the effects of exogenous GAs (GA_3_), CKs (6-Benzylaminopurine, BA), and the inhibitor of GA biosynthesis, uniconazole, on *L*/*R*, LMA, N_area_, photosynthetic traits, and whole-plant RGR in seedlings of *Polygonum cuspidatum* grown under two light intensities, each at two nitrogen availabilities (Sugiura et al., 2015a). We revealed that morphology, physiology, and whole-plant RGR were largely altered by these substances. Furthermore, the seedlings with high levels of endogenous GAs or CKs showed morphologies similar to those treated with GA_3_ or BA, respectively. The seedlings grown at low N availability with low levels of GAs and CKs were similar to those treated with uniconazole. Previously, genotypic variability in GA levels was argued to be responsible for variations in biomass allocation (Rood et al., 1983; Dijkstra et al., 1990; Lambers et al., 1995; Nagel et al., 2001a; Bultynck and Lambers, 2004). Additionally, our previous results clearly suggest that not only CKs (Takei et al., 2001; Sakakibara, 2003; Rahayu et al., 2005), but also GAs are responsible for the phenotypic variation of biomass allocation in response to nitrogen availability.

We also proposed a schematic model for the regulatory mechanisms of biomass allocation and whole-plant RGR based on the results of our previous study. Briefly, endogenous levels of GAs and CKs are both determined by nitrogen availability, while GAs are additionally regulated by light intensity. Both GAs and CKs affect biomass allocation, while GAs also affect LMA and thereby leaf area ratio (the proportion of leaf area to total plant biomass, LAR, m^2^ g^−1^). These morphological changes lead to changes in nitrogen acquisition, which is important for the production of Rubisco, a photosynthetic protein, and therefore eventually result in changes in the net assimilation rate (NAR, g m^−2^ d^−1^). Thus, LAR and NAR determine the whole-plant RGR because RGR is the product of LAR and NAR (Sugiura et al., 2015a).

However, the model remains to be confirmed because we conducted the quantification of GAs and CKs under only a few stable conditions and at the whole-shoot level in the previous study, causal relationships between changes in biomass allocation and the levels of GAs and CKs were still unclear. Moreover, new leaves are produced in the shoot apex, and morphological and physiological characteristics of new leaves are affected by environmental variables sensed not only by these developing leaves, but also by mature leaves (Yano and Terashima, 2001; Miyazawa et al., 2011; Munekage et al., 2015). Therefore, it is necessary to analyse relationships among changes in biomass allocation, morphological and physiological traits, and the levels of hormones, not at the whole-shoot level, but at each leaf and shoot apex.

The role of GAs in the regulation of LMA also remains unsolved. LMA consists of not only structural components such as cell wall, but also non-structural components such as soluble sugars and starch (total non-structural carbohydrate, TNC; Poorter et al., 2009). It is also known that LMA is increased in *Polygonum* species grown under high CO_2_ concentration and low nitrogen availability, which is due to the accumulation of both structural components and TNC in the leaves (Ishizaki et al., 2003; Onoda et al., 2007). Thus, it is probable that endogenous GAs regulate LMA by controlling both structural and non-structural carbohydrates in the leaves.

The first purpose of this study is to reveal the causal relationships among changes in biomass allocation, nitrogen availability, and light intensity and levels of GAs and CKs in *P. cuspidatum*. Our hypothesis is that the levels of hormones such as GAs and CKs would change in response to drastic changes in environmental variables, which would cause changes in biomass allocation at the whole-plant level. The second purpose is to consider the regulatory mechanisms of biomass allocation by GAs and CKs in a detailed manner from the separate analysis of phytohormones in the shoot apex and each leaf. Since shoot apices and young leaves are sink organs for photosynthates, we hypothesized that changes in the levels of the phytohormones that regulate their sink activities would correspond to changes in biomass allocation at the whole-plant level. The third purpose is to elucidate the roles of phytohormones in the regulation of morphological and physiological traits of leaves such as LMA and photosynthetic traits. GAs may be involved in the regulation of LMA as mentioned above, and CKs are also involved in the regulation of the photosynthetic rate through the accumulation of nitrogen and retardation of leaf senescence (Boonman et al., 2007, 2009). We hypothesized that, in each individual leaf, the reduction in the levels of GAs would cause the accumulation of TNC and structural components, which would increase their LMAs. We also hypothesized that a reduction in the levels of CKs would cause a decrease in leaf nitrogen content, which would decrease the photosynthetic rate of each individual leaf.

In the present study, in order to reveal the roles of GAs and CKs in a detailed manner, seedlings of *P. cuspidatum* were grown under various light and nitrogen conditions and subjected to various experimental treatments to cause changes in biomass allocation and morphological and physiological traits of leaves.

## Materials and methods

### Plant materials

**Figure 1:**
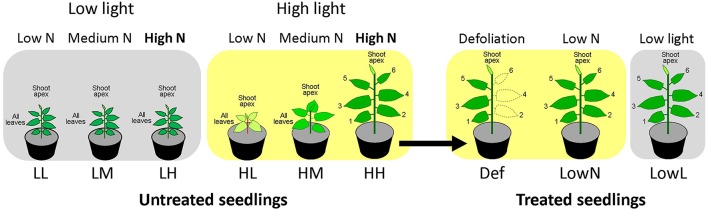
**The experimental design of the present study**. Seedlings of *Polygonum cuspidatum* were grown under low light and low nitrogen (LL), low light and medium nitrogen (LM), low light and high nitrogen (LH), high light and low nitrogen (HL), high light and medium nitrogen (HM), and high light and high nitrogen (HH) conditions for about 51 to 55 days after sowing, and they were termed “untreated seedlings.” HH seedlings were further defoliated (Def) or transferred to low nitrogen (LowN) or low light (LowL) conditions and termed “treated seedlings”, and they were grown for 7–9 days. In those seedlings, the oldest leaf and subsequent leaves were referred to as leaf 1, leaf 2, leaf 3, leaf 4, leaf 5, and leaf 6, respectively. Endogenous levels of phytohormones were quantified in shoot apex and all leaves in LL, LM, LH, HL, and HM seedlings, and in shoot apex and each leaf in HH, Def, LowN, and LowL seedlings (see text in detail).

The experimental design was described in Figure 1. We used seeds of *P. cuspidatum* collected on Mt Fuji in 2007. They were grown under six constant growth conditions in growth rooms: Low light and low nitrogen (LL), low light and medium nitrogen (LM), low light and high nitrogen (LH), high light and low nitrogen (HL), high light and medium nitrogen (HM), and high light and high nitrogen (HH) conditions, and the seedlings grown under the constant conditions are hereafter called “untreated seedlings”. The light and dark cycle was 12/12 h. Light was provided by an array of LEDs (NSPWR70CS-K1, Nichia, Tokushima, Japan) with peak wavelengths at 440 and 550 nm. Photosynthetically active photon flux densities (PFDs) for high and low light were 250 and 80 μmol m^−2^ s^−1^. Air temperature and relative humidity were about 24°C and 50%, respectively. The nutrient solution contained 1 mM NaH_2_PO_4_, 0.25 mM Na_2_HPO_4_, 1 mM MgSO_4_, 10 μM Fe-EDTA, 100 μM MnSO_4_, 300 μM H_3_BO_3_, 10 μM ZnSO_4_, 1 μM CuSO_4_, 0.25 μM (NH_4_)_6_Mo_7_O_24_, and 1.25 μM CoCl_2_. Low (0.1 mM), medium (1 mM), and high nitrogen (5 mM) solutions with 2 mM K and Ca concentrations were obtained by adding KNO_3_, Ca (NO_3_)_2_, KCl, and CaCl_2_.

The seeds were sown in plastic pots (5.5 cm in height and 6 cm in diameter for LL, LM, and LH, and 7.5 cm in height and 9 cm in diameter for HL, HM, and HH) filled with river sand. We used smaller pots for LL, LM, and LH, since they have fewer roots than HL, HM, and HH. After germination, 20 mL of the nutrient solution was applied to each pot every other day. When necessary, the pots were watered to keep the soil moist. LL, LM, and LH seedlings were grown for 55 days after sowing. HL, HM, and HH seedlings were grown for 51~53 days after sowing. The oldest leaf and subsequent leaves, having leaf areas greater than 5 cm^2^, were referred to as leaf 1, leaf 2, leaf 3, leaf 4, leaf 5, and leaf 6, respectively, in HH seedlings.

### The treatments

Fifty-three days after sowing, some of the HH seedlings were defoliated (Def) or transferred to low nitrogen (LowN) or low light (LowL) conditions and termed “treated seedlings” (Figure 1). In Def, every other leaf was defoliated from the stem to reduce the cumulated area of the leaves to about half. For LowN transfer, the remaining nutrients in the soil were flushed away using tap water and a nutrient solution containing 0.1 mM N was applied. LowL seedlings were transferred to the low light conditions (80 μmol m^−2^ s^−1^). The number of plants for each treatment was four to eight, and they were grown for 79 days after the treatments.

### Photosynthesis measurements

The photosynthetic rate of the leaf was measured using a portable gas-exchange system (GFS-3000, Walz, Effeltrich, Germany) on the day before the leaf and whole-plant sampling (see below). That is, 54 days after sowing for LL, LM, and LH seedlings, 50~52 days after sowing for HH seedlings, and 58~60 days after sowing for Def, LowN, and LowL seedlings. New leaves that emerged after the onset of the treatments were referred to as leaf 7 and leaf 8 and were also subjected to the measurements in Def, LowN, and LowL seedlings. The measurements were conducted using the leaves of four to eight HM, LL, LM, or LH seedlings (one leaf per seedling) and for five or six mature leaves for each of four to eight HH, Def, LowN, or LowL seedlings. The CO_2_ concentration, leaf temperature, relative humidity, and vapor pressure deficit in the chamber were 400 μmol mol^−1^, 25°C, 50%, and 1.45–1.6 kPa, respectively. The rate of photosynthesis under the growth conditions (A_growth_) was measured at a PFD of 250 μmol m^−2^ s^−1^ for HM and HH and 80 μmol m^−2^ s^−1^ for LL, LM, and LH. The maximum photosynthetic rate (A_max_) was measured at a PFD of 1000 μmol m^−2^ s^−1^ for HM and HH and 500 μmol m^−2^ s^−1^ for LL, LM, and LH. For HL seedlings, we did not measure A_max_ or A_growth_, since the leaves were too small for accurate measurement. A_growth_ values were also calculated at the whole-leaf level by weighted-averaging all of the values from the younger to the older leaves for HH, Def, LowN, and LowL.

### Sampling

In the middle of the light period on the following day of the photosynthesis measurements, seedlings were harvested for biochemical and growth analyses. Around 50 mg of shoot apices and 100 mg of leaves in fresh weight were required for the quantifications of the hormones, so two to six shoot apices and 6 to 10 leaf disks (1 cm in diameter) were pooled as one replicate for LL, LM, LH, HL, and HM seedlings. One shoot apex and three leaf disks from different leaves were obtained as one replicate for HH, Def, LowN, and LowL seedlings. The number of replicates for each of the growth conditions or treatments was four to eight. The samples for the hormone quantifications were immediately weighed, frozen in liquid N_2_, and stored at −80°C. After the sampling, plants were divided into leaves, stems, and roots, and the remaining leaves were scanned using a flatbed scanner (CanoScan LiDE 210; Canon, Tokyo, Japan) to determine the leaf area. The number of leaves was also recorded for each seedling. They were oven-dried at 80°C for more than 2 days.

### Growth analysis

The *L*/*R* and LMA were calculated from the dry mass of these samples. The leaf biomass used for the quantification of endogenous hormones was estimated from the number of leaf disks and the LMA of the remaining leaves. The leaf biomass, leaf area, and LMA were obtained for each leaf in the HH, Def, LowN, and LowL seedlings. Oven-dried samples were ground using a Multi-beads Shocker (Yasui Kikai, Osaka, Japan) and the nitrogen content of the ground samples were measured using a CN elemental analyser (Vario EL, Elementar Analyzensysteme GmbH, Hanau, Germany). We obtained the nitrogen content per mass (N_mass_, g N g^−1^) for the leaves, stems, and roots. The N_area_ was calculated as a product of the N_mass_ and LMA in each leaf. The plant nitrogen concentration (PNC, g N g^−1^) and carbon-to-nitrogen ratio (C/N ratio, g C g N^−1^) were calculated from the carbon content per mass (g C g^−1^) and N_mass_ for the leaves, stems, and roots. The nitrate content of each leaf in the HH, Def, LowN, and LowL seedlings was analyzed following Cataldo et al. (1975). The LMA and N_area_ were also calculated at the whole-leaf level by weighted-averaging all of the values from the youngest to the oldest leaves in HH, Def, LowN, and LowL.

To evaluate the changes in the biomass allocation between the leaves and roots after the treatments, we defined Δ*L*/Δ*R* (g g^−1^) as the ratio of the differences in leaf biomass and those in root biomass between the treated and untreated seedlings as follows:

$$\begin{array}{l} \Delta L/\Delta R\;=\;\frac{L_{Treated}/L_{HH}}{R_{Treated}/R_{HH}} \end{array}$$

where *L*_*Treated*_ and *R*_*Treated*_ are the leaf and root biomasses of the treated seedlings (Def, LowN, and LowL), and *L*_*HH*_ and *R*_*HH*_ are the leaf and root biomasses of the HH seedlings. Δ*L*/Δ*R* of the untreated seedlings, LL, LM, LH, HL, HM, and HH, were equal to *L*/*R* assuming that they did not change biomass allocation between the leaves and roots and kept constant *L*/*R* under constant growth conditions, based on a previous study (Osone and Tateno, 2005). Δ*L*/Δ*R* can be a better indicator of changes in biomass allocation than *L*/*R* since it can highlight the changes clearly.

### Total non-structural carbohydrate analysis

The contents of glucose, sucrose, and starch (TNC) of the leaves, stems, and roots were determined using ground samples as described in Sugiura et al. (2015b). Soluble sugars were extracted using 80%ethanol at 80°C, and the precipitate was used for the determination of starch. Sucrose was hydrolysed to glucose and fructose by the invertase solution (Wako Chemicals, Tokyo, Japan). Starch was broken down to glucose by amyloglucosidase (A-9228; Sigma-Aldrich, St Louis, MO). The glucose content and glucose equivalents of sucrose and starch were quantified using a Glucose CII test kit (Wako Chemicals) and TNC was calculated on a mass basis and also on leaf area basis. Structural LMA (g m^−2^) was calculated using the difference between LMA and TNC (Bertin and Gary, 1998; Sugiura et al., 2015b). TNC was further calculated at the whole-leaf level by weighted-averaging all of the values from the youngest to the oldest leaves in HH, Def, LowN, and LowL.

### Quantification of endogenous GAs and CKS using ultra-performance liquid chromatography–electrospray ionisation–tandem quadruple mass spectrometry

The frozen samples were used for the extraction and quantification of phytohormones using an LC-MS system (Kojima et al., 2009). Endogenous levels of GA_12_, GA_24_, GA_9_, GA_53_, GA_44_, GA_19_, GA_20_, GA_1_, and GA_8_ were quantified and the sum of them was defined as the endogenous level of GAs. Endogenous levels of the trans-zeatin type cytokinins (tZs) and those of the N^6^-(Δ^2^-isopentenyl) adenine type (iPs) were quantified and the sum of them was defined as the endogenous level of CKs. They were shown separately, since tZs are mainly synthesized in roots and transported to shoots, whereas iPs are mainly synthesized in shoots (Matsumoto-Kitano et al., 2008; Kudo et al., 2010). Endogenous levels of abscisic acid (ABA), indole-3-acetic acid (IAA), salicylic acid (SA), and jasmonic acid (JA) were also determined. Those quantified in the shoot apex and leaves were calculated on a fresh-weight basis and leaf-area basis, respectively. The endogenous levels of phytohormones were also calculated at the whole-leaf level by weighted-averaging all of the values from the youngest to the oldest leaves in HH, Def, LowN, and LowL.

### Statistical analysis

The morphological and physiological traits and the endogenous levels of hormones were compared among the untreated seedlings in low light (LL, LM, and LH) and high light (HL, HM, and HH) by one-way ANOVA followed by multiple pairwise Tukey's test comparisons. Those of the treated seedlings (Def, LowN, and LowL) were also compared with HH using a Student's *t*-test (Systat13, Hulinks Inc., Tokyo, Japan). Note that it is assumed that these traits would not change so much in 79 days in HH seedlings although the ages of HH seedlings (50~52 days-old) and Def, LowN, and LowL seedlings (58~60 days-old) were different.

## Results

### Morphological traits, photosynthetic traits, and TNC at the whole-plant level

**Figure 2:**
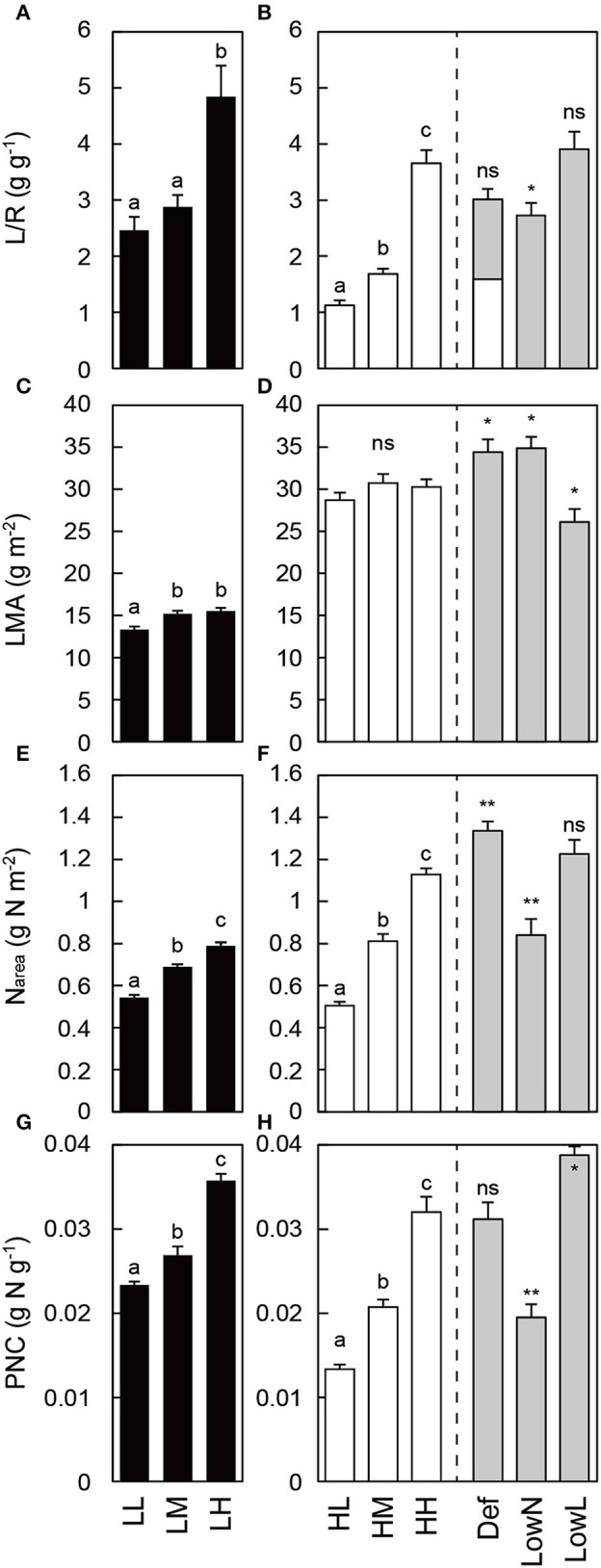
**Morphological traits and nitrogen content in ***Polygonum cuspidatum*** grown under low light (A,C,E,G) and high light conditions (B,D,F,H)**. See text for abbreviations. For LMA and N_area_, values obtained from the all leaves are presented for LL, LM, LH, HL, and HM, and weighted mean values of all the leaves are resented for HH, Def, LowN, and LowL. The white bar inside the value of L/R in Def **(B)** represents the value just after the defoliation treatment. Letters indicate significant differences among nitrogen treatments in low light (LL, LM, and LH) and high light (HL, HM, and HH; one-way ANOVA followed by, *P* < 0.05, Tukey's test, *P* < 0.05). Asterisks indicate significant differences in Def, LowN, and LowL compared with HH (Student's *t*-test, ^*^P < 0.05; ^**^P < 0.01). Values are means + SE (*n* = 4–8).

Morphological traits differed in response to light intensity and nitrogen availability in the untreated seedlings (LL, LM, LH, HL, HM, and HH seedlings) grown under constant conditions. *L*/*R* calculated from the dry mass of each organ (Figures S1A,B) was higher in low light than in high light, and it increased with nitrogen availability (Figures 2A,B). The number of leaves increased with nitrogen availability under both light conditions (Figures S1C,D). The LMA was almost constant among the untreated seedlings under both light conditions (Figures 2C,D). The N_area_ and PNC consistently differed with nitrogen availability in the untreated seedlings (Figures 2G,H). These traits in HH were significantly altered after the treatments (Def, LowN, and LowL). Although *L*/*R* was reduced to half by the defoliation in Def, it recovered to almost the same level as that in HH 79 days after the defoliation. *L*/*R* decreased significantly in LowN and did not change significantly in LowL (Figure 2B). LMA increased in Def and LowN and decreased in LowL (Figure 2D). N_area_ increased in Def, decreased in LowN, but did not change in LowL (Figure 2F). PNC did not change in Def, decreased in LowN, and increased in LowL (Figure 2H).

**Figure 3:**
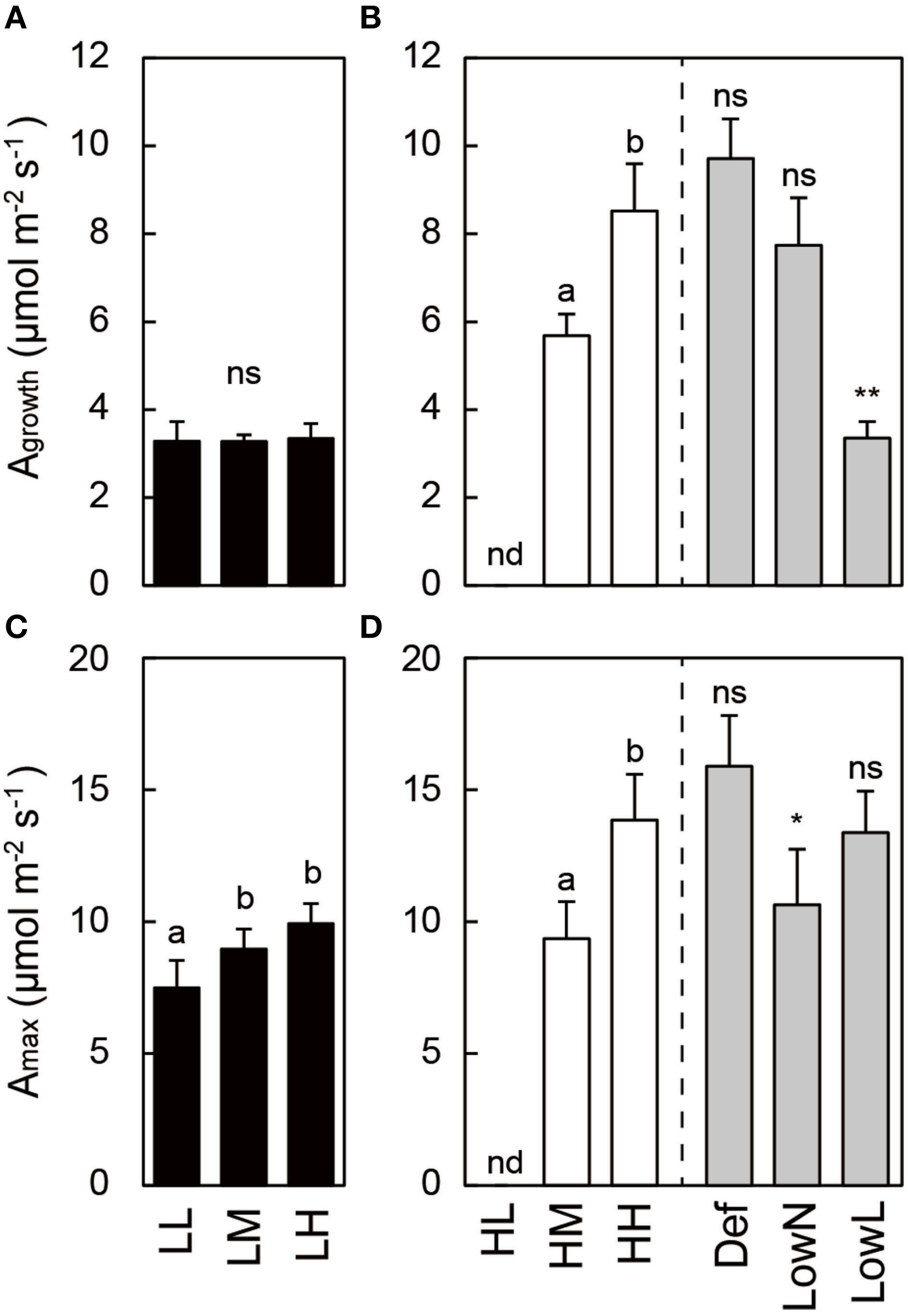
**Ambient photosynthetic rate (A_**growth**_; A,B) and maximum photosynthetic rate (A_**max**_; C,D) in ***Polygonum cuspidatum*****. See text for abbreviations. Values obtained from the all leaves are presented for LL, LM, LH, HL, and HM, and weighted mean values of all the leaves are presented for HH, Def, LowN, and LowL. Values in HL were not obtained due to small leaf size. A_growth_ was measured at 80 μmol m^−2^ s^−1^ in LL, LM, LH, and LowL and at 250 μmol m^−2^ s^−1^ in HL, HM, HH, Def, and LowN. Letters indicate significant differences among nitrogen treatments in low light (LL, LM, and LH) and high light (HL, HM, and HH) (one-way ANOVA, *P* < 0.05, followed by Tukey's test, *P* < 0.05). Asterisks indicate significant differences in Def, LowN, and LowL compared with HH (Student's *t*-test, ^*^P < 0.05; ^**^P < 0.01). nd means no data. Values are means + SE (*n* = 4–8).

Photosynthetic traits also differed in response to light intensity and nitrogen availability in the untreated seedlings. A_growth_ was lower in low light than in high light, and A_max_ increased with nitrogen availability in each light condition in the untreated seedlings (Figures 3A–D). A_growth_ and A_max_ in HH were altered by the treatments. The decrease in A_growth_ in LowL was due to the decrease in the growth PFD from 250 to 80 μmol m^−2^ s^−1^. A_max_ was significantly decreased only in LowN (Figure 3D).

**Figure 4:**
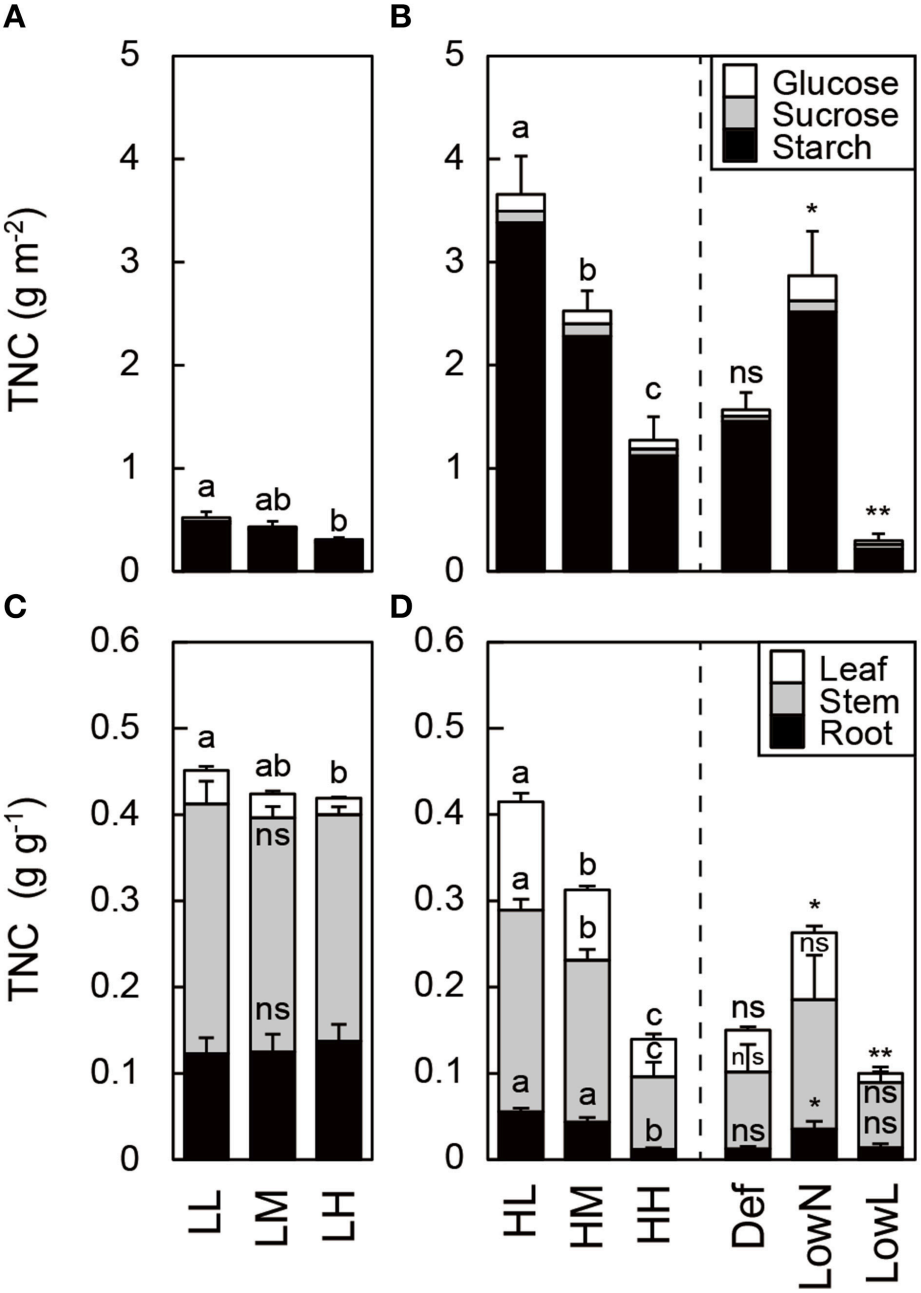
**Total non-structural carbohydrates (TNC) in leaves (A,B) and in each organ (C,D) in ***Polygonum cuspidatum*****. See text for abbreviations. Bars from top to bottom represent values of glucose (white bar), sucrose (gray bar), and starch (black bar) in **(A,B)**. Bars from top to bottom represent values of TNC in leaves (white bar), stems (gray bar), and roots (black bar) in **(C,D)**. Values obtained from the all leaves are presented for LL, LM, LH, HL, and HM, and weighted mean values of all the leaves are presented for HH, Def, LowN, and LowL. Letters indicate significant differences among nitrogen treatments in low light (LL, LM, and LH) and high light (HL, HM, and HH; one-way ANOVA, *P* < 0.05, followed by Tukey's test, *P* < 0.05). Asterisks indicate significant differences in Def, LowN, and LowL compared with HH (Student's *t*-test, ^*^P < 0.05; ^**^P < 0.01). Values are means + SE (*n* = 4–8).

TNC differed in response to light intensity and nitrogen availability in the untreated seedlings. Starch comprised more than 90% of TNC in all organs in all of the seedlings (Figures 4A,B). TNC in leaves was lower in low light than in high light, and it decreased with nitrogen availability (Figures 4A–D). After the treatments, TNC was increased in LowN, decreased in LowL, but did not change in Def compared with HH (Figures 4B,D).

### Endogenous levels of GAs and CKS in the shoot apex and all leaves

We used the level of GAs, which is the level of sum of GA precursors and biologically active GA, as a representative value to discuss the association of them with the morphological and physiological traits. This is because the level of precursors reflects the level of GA_1_ (Figure S2) as reported in previous studies (Dayan et al., 2012; Regnault et al., 2015), and GA_1_ was not detected in the leaves of HL (Table 1) and in the older leaves with low levels of GAs in HH, Def, LowN, and LowL.

**Table 1:** **Endogenous levels of precursors and activated forms of GAs in the all leaves of LL, LM, LH, HL, and HM and the youngest leaves of HH, Def, LowN and LowL in ***Polygonum cuspidatum*****.

**Whole or youngest leaves**									
**nmol m**^−2^	**GA**_12_	**GA**_24_	**GA**_9_	**GA**_53_	**GA**_44_	**GA**_19_	**GA**_20_	**GA**_1_	**GA**_8_
LL	n.d	n.d	n.d	1.27 ± 0.42	0.18 ± 0.10	0.74 ± 0.14	0.12 ± 0.02	0.26 ± 0.15	2.42 ± 0.37
LM	n.d	n.d	n.d	1.96 ± 1.13	0.51 ± 0.46	1.13 ± 0.37	0.14 ± 0.02	0.30 ± 0.09	3.08 ± 0.60
LH	n.d	n.d	n.d	3.31 ± 0.95	0.50 ± 0.04	2.09 ± 0.53	0.19 ± 0.03	0.99 ± 0.09	5.14 ± 0.56
HL	n.d	0.23 ± 0.22	n.d	11.5 ± 1.8	0.54 ± 0.12	4.26 ± 0.49	0.13 ± 0.02	n.d	1.29 ± 0.16
HM	n.d	0.02 ± 0.01	n.d	15.2 ± 1.5	0.45 ± 0.19	6.17 ± 0.39	0.15 ± 0.01	0.34 ± 0.02	2.69 ± 0.22
HH	n.d	0.11 ± 0.03	n.d	91.9 ± 13.2	5.18 ± 0.95	19.90 ± 2.86	0.36 ± 0.09	1.79 ± 0.24	6.37 ± 1.15
Def	n.d	0.07 ± 0.02	n.d	149.3 ± 25.9	6.18 ± 1.88	25.16 ± 1.43	0.33 ± 0.09	1.49 ± 0.17	4.56 ± 1.01
LowN	n.d	n.d	n.d	27.5 ± 9.1	0.44 ± 0.17	12.54 ± 1.37	0.20 ± 0.02	0.53 ± 0.12	2.10 ± 0.45
LowL	n.d	n.d	n.d	43.2 ± 6.1	3.65 ± 1.09	22.10 ± 3.14	0.22 ± 0.04	0.98 ± 0.11	4.34 ± 0.93

*GA_12_, GA_24_, GA_9_, GA_53_, GA_44_, GA_19_, and GA_20_ are precursor forms of GA_1_ and GA_8_ is inactivated form of GA_1_. Values are means ± SE (n = 4–8). n.d means not detectable*.

Differences in the endogenous levels of GAs and CKs were found in both the shoot apex and leaves in the untreated seedlings. The levels of GAs and CKs increased in both the shoot apex and leaves with nitrogen availability (Figures S3A–H). After the treatments, the levels of GAs in the shoot apex increased in LowL, decreased in LowN, and did not change significantly in Def (Figure S3B), while the levels of CKs in the shoot apex were similar across the treatments (Figure S3D). The levels of GAs in the leaves increased in Def, decreased in LowN, and did not change in LowL (Figure S3F), and that of CKs in the leaves decreased only in LowN (Figure S3H).

### Relationships between levels of GAs and CKS in leaves and shoot apex and biomass allocation between leaves and roots

**Figure 5:**
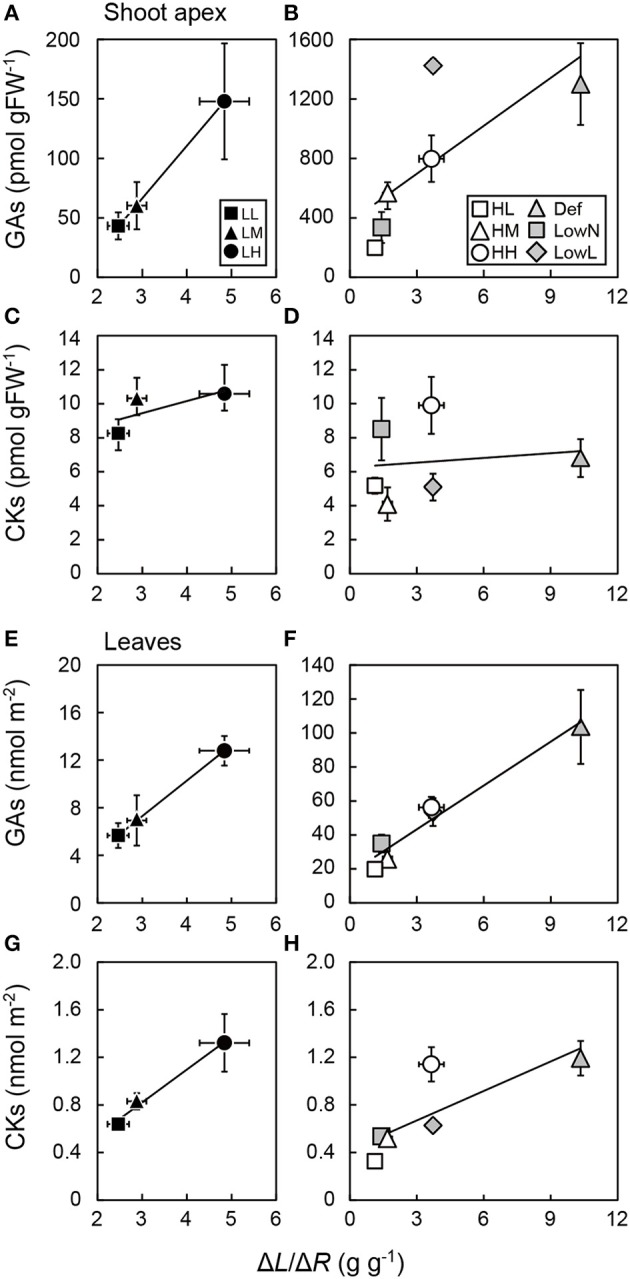
**Relationships between endogenous levels of GAs and CKs in the shoot apex (A–D) and leaves (E–H) and the ratio of the differences in leaf biomass and root biomass (Δ***L***/Δ***R***) in ***Polygonum cuspidatum*****. See text for abbreviations. For GAs and CKs in leaves, values obtained from the all leaves are presented for LL, LM, LH, HL, and HM, and weighted mean values of all the leaves are presented for HH, Def, LowN, and LowL. Closed rectangles, triangles, and circles denote LL, LM, and LH, and open rectangles, triangles, and circles denote HL, HM, and HH, respectively. Gray triangles, rectangles, and diamonds denote Def, LowN, and LowL, respectively. Solid lines represent regression lines; **(A)** *R*^2^ = 0.99, **(B)** *R*^2^ = 0.54, **(C)** *R*^2^ = 0.51, **(D)** *R*^2^ = 0.02, **(E)** *R*^2^ = 1, **(F)** *R*^2^ = 0.96, **(G)** *R*^2^ = 0.98, **(H)** *R*^2^ = 0.64. Values are means ± SE (*n* = 4–8).

After the treatments, Δ*L*/Δ*R*, which shows the changes in biomass allocation between leaves and roots, increased largely in Def, decreased largely in LowN, and did not change in LowL compared with HH seedlings (Figure 5). The levels of GAs in the shoot apex were highly correlated with Δ*L*/Δ*R* in the untreated seedlings in low light conditions (Figure 5A) and in the untreated and treated seedlings in high light conditions (Figure 5B). Although Δ*L*/Δ*R* was lower in LowL than in Def, the levels of GAs in the shoot apex were highest in LowL. The levels of CKs in the shoot apex were poorly correlated with Δ*L*/Δ*R* in the untreated seedlings in low light conditions (Figure 5C) and in the untreated and treated seedlings in high light conditions (Figure 5D).

The levels of GAs and CKs in the leaves were highly correlated with Δ*L*/Δ*R* in the untreated seedlings in low light conditions (Figures 5E,G) and in the untreated and treated seedlings in high light conditions (Figure 5F), while the levels of CKs in the leaves were less correlated with Δ*L*/Δ*R* in the untreated and treated seedlings in high light conditions (Figure 5H).

### Endogenous levels of phytohormones in each part of the seedlings

**Figure 6:**
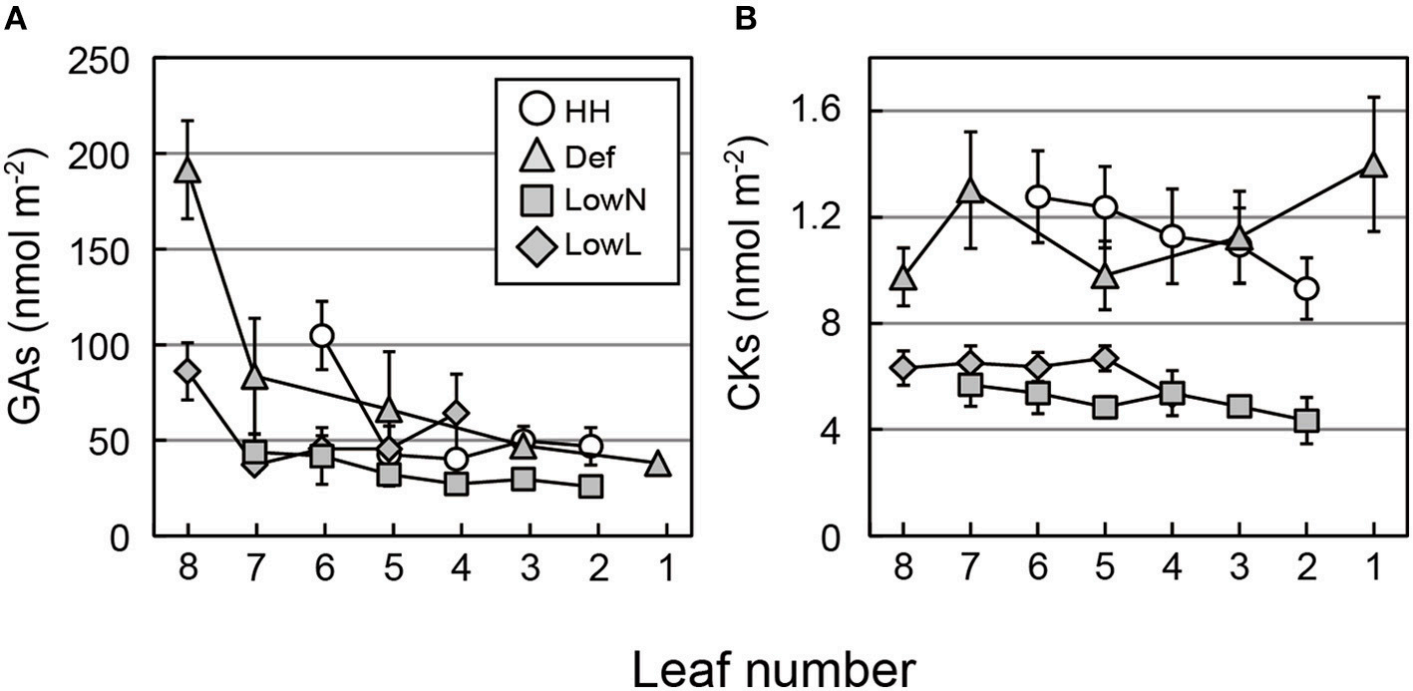
**Endogenous levels of GAs (A) and CKs (B) in each leaf of ***Polygonum cuspidatum*****. See text for abbreviations. Open circles, gray triangles, gray rectangles, and gray diamonds denote HH, Def, LowN, and LowL, respectively. Numbers on the *x*-axes denote the leaf position (see text for details). Values are means ± SE (*n* = 4–8).

The levels of GAs were highest in the youngest leaves and decreased with leaf age in all of the seedlings (Figure 6A). The levels of CKs did not differ markedly with leaf age in all of the seedlings, but they were higher in HH and Def than those in LowN and LowL (Figure 6B). The levels of tZs were highest in the youngest leaves and decreased with leaf age in all of the seedlings except for LowL (Figure S4C). The changes in the levels of iPs were almost the same as those of CKs (Figure S4D). Consistent trends were not observed in the levels of IAA and ABA among the treatments or among the leaves (Figures S4E,F), whereas those of SA and JA were highest in the youngest leaves and decreased with leaf age (Figures S4G,H).

We compared the relationships between the levels of GAs and CKs in the shoot apex and those in the leaves to get an insight into the transport pathway of GAs and CKs from the leaves to the shoot apex. Values obtained from the all leaves were used in LL, LM, LH, HL, and HM, and those obtained from the youngest leaves were used in HH, Def, LowN, and LowL. There were strong positive correlations between the levels of GAs in the shoot apex and those in the leaves under both low and high light conditions (Figures S5A,B), although the levels of GAs in the shoot apex in LowL were the highest. The correlation between the level of CKs in the shoot apex and those in the leaves was lower than the correlation between the level of GAs in the shoot apex and that in the leaves (Figures S5C,D).

We also found differences in the levels of activated and inactivated GAs in the shoot apex and in the all leaves or the youngest leaves among the untreated and treated seedlings (Tables 1, 2). The level of GA_1_, a biologically active gibberellin, is much lower than those of GA precursors, and the differences in the levels of GAs were mostly due to the differences in the level of GA_53_, especially in high light conditions, the levels of GA precursors. However, the level of GA_1_ was well correlated with the level of sum of GA precursors in both the shoot apex and leaves under both light conditions (Figure S2).

**Table 2:** **Endogenous levels of precursors and activated forms of GAs in the shoot apex in ***Polygonum cuspidatum*****.

**Shoot apex**									
**pmol gFW**^−1^	**GA**_12_	**GA**_24_	**GA**_9_	**GA**_53_	**GA**_44_	**GA**_19_	**GA**_20_	**GA**_1_	**GA**_8_
LL	n.d	n.d	n.d	13.47 ± 5.41	3.93 ± 1.37	16.48 ± 4.19	1.92 ± 0.27	1.12 ± 0.01	6.83 ± 1.11
LM	n.d	n.d	n.d	19.45 ± 7.34	8.35 ± 4.91	17.35 ± 4.61	2.62 ± 0.62	4.12 ± 0.32	10.84 ± 3.27
LH	n.d	n.d	n.d	61.98 ± 31.79	21.02 ± 4.60	30.22 ± 10.14	3.60 ± 0.30	7.28 ± 1.55	23.75 ± 3.95
HL	n.d	1.20 ± 1.05	n.d	86.5 ± 15.6	21.7 ± 2.8	40.8 ± 7.0	2.81 ± 0.15	4.19 ± 0.02	10.51 ± 1.87
HM	n.d	0.35 ± 0.26	n.d	266.2 ± 97.7	27.2 ± 11.6	40.0 ± 6.6	1.47 ± 0.42	3.65 ± 1.26	14.18 ± 2.32
HH	n.d	0.39 ± 0.02	54.2 ± 52.6	543.7 ± 114.7	66.0 ± 11.2	85.0 ± 10.0	2.50 ± 0.20	6.09 ± 1.36	25.95 ± 3.60
Def	n.d	n.d	n.d	1043.8 ± 284.0	60.6 ± 4.4	100.5 ± 15.2	1.92 ± 0.54	5.71 ± 0.83	16.28 ± 2.48
LowN	n.d	n.d	39.8 ± 19.6	226.3 ± 93.0	8.8 ± 1.9	78.2 ± 8.8	1.93 ± 0.33	2.50 ± 0.70	5.11 ± 0.78
LowL	n.d	n.d	42.0 ± 4.7	1128.7 ± 45.7	71.0 ± 19.2	147.4 ± 24.8	1.25 ± 0.19	7.74 ± 1.13	25.29 ± 7.81

*GA_12_, GA_24_, GA_9_, GA_53_, GA_44_, GA_19_, and GA_20_ are precursor forms of GA_1_ and GA_8_ is inactivated form of GA_1_. Values are means ± SE, n = 4–8*.

### Leaf morphology, photosynthetic traits, and TNC in each leaf

**Figure 7:**
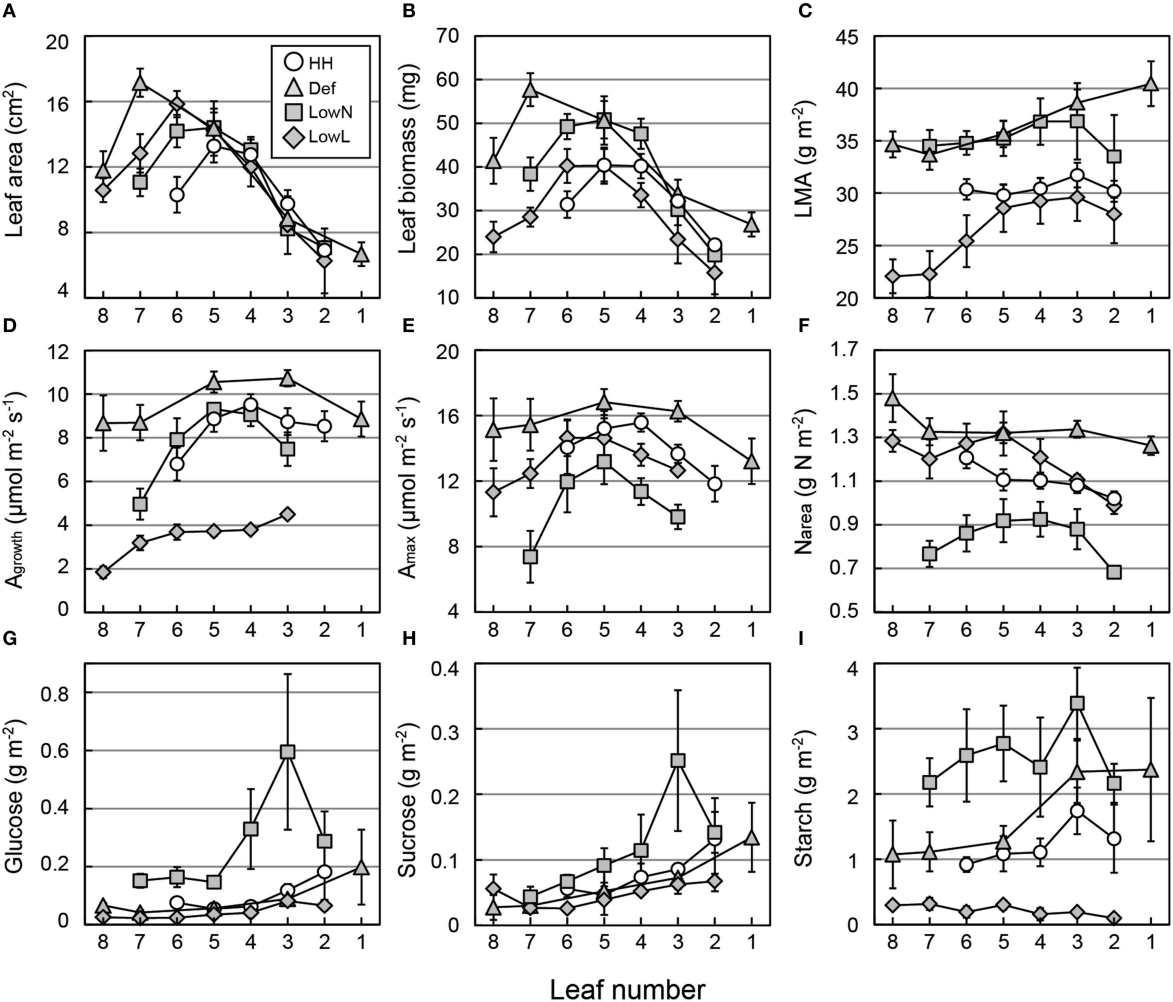
**Leaf morphology (A–C), photosynthetic rate (D,E), leaf nitrogen content per area (F), and soluble sugars and starch (G–I) in each leaf of ***Polygonum cuspidatum*****. See text for abbreviations. Open circles, gray triangles, gray rectangles, and gray diamonds denote HH, Def, LowN, and LowL, respectively. Numbers on the *x*-axes denote the leaf position (see text for details). A_growth_ and A_max_ were not measured in leaf 2 in LowN and LowL, while leaf 1 was additionally measured in Def. Values are means ± SE (*n* = 4–8).

Age-dependent changes in the morphology, photosynthetic traits, and TNC of each leaf were investigated (Figure 7). After the onsets of the treatments, the leaf area was almost stable between leaf 2 and leaf 5 (Figure 7A), whereas the leaf biomass of these leaves changed (Figure 7B). Consequently, the LMA of leaf 2 to leaf 5 increased in Def and LowN and slightly decreased in LowL (Figure 7C). The leaf area and leaf biomass of the leaves produced after the onset of the treatments, leaf 7 and leaf 8, were greater in Def than those in LowN and LowL. The LMA values of these leaves were similar to those of the older leaves (leaf 1 to leaf 5).

The decrease in A_growth_ in LowL was due to reduced PFD as mentioned above, and the increase in A_growth_ was observed in Def (Figure 7D). An increase in A_max_ and N_area_ in Def and a decrease in A_max_ and N_area_ in LowN were observed in all leaves (Figures 7E,F). The changes in A_growth_ and A_max_ were mostly consistent with the changes in N_area_ (Figure 7F). Changes in the N_mass_ and nitrate content of the leaf were almost consistent with those in N_area_ (Figures S4A,B).

The TNC contents of individual leaves were further investigated. Glucose and sucrose contents tended to increase with age in all of the seedlings (Figures 7G,H). Starch contents also tended to increase with the leaf age, except for those in LowL (Figure 7I).

### Interrelationships among leaf morphological and physiological traits, TNC, and endogenous phytohormones

**Figure 8:**
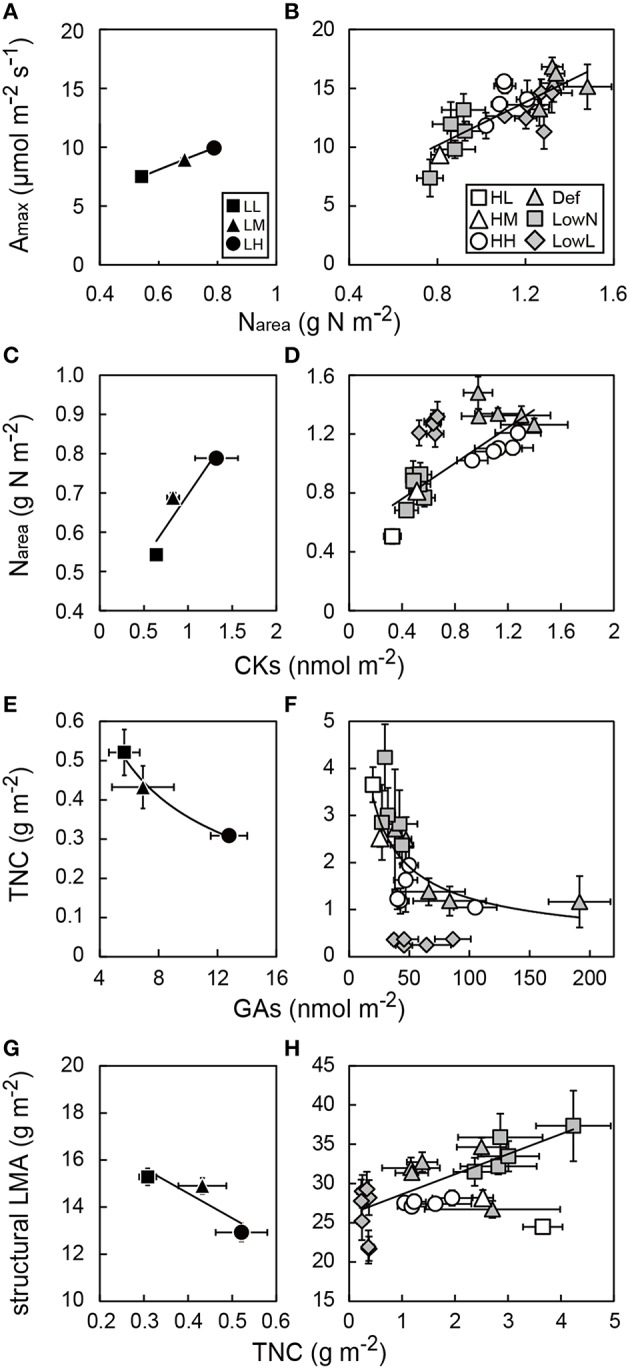
**Relationships between maximum photosynthetic rate (A_**max**_) and leaf nitrogen content per area (N_**area**_), between N_**area**_ and endogenous levels of CKs in leaves, between total non-structural carbohydrates (TNC) and endogenous levels of GAs, and between structural leaf mass per area (sLMA) and TNC in low light (A,C,E,G) and high light conditions (B,F,F,H) in ***Polygonum cuspidatum*****. See text for abbreviations. For GAs, N_*area*_ TNC, and structural LMA in leaves, values obtained from the all leaves are presented for LL, LM, LH, HL, and HM, and those from each leaf are presented for HH, Def, LowN, and LowL. Closed rectangles, triangles, and circles denote LL, LM, and LH, and open rectangles, triangles, and circles denote HL, HM, and HH, respectively. Gray triangles, gray rectangles, and gray diamonds denote Def, LowN, and LowL, respectively. Solid lines and curves represent regression lines and curves for LL, LM, and LH **(A,C,E,G)** and HL, HM, HH, Def, and LowN **(B,F,F,H)** where the value of LowL and those of HL, HM, and HH were excluded in **(D,F)** and **(H)**, respectively; **(A)** *R*^2^ = 0.81, **(B)** *R*^2^ = 0.60, **(C)** *R*^2^ = 0.88, **(D)** *R*^2^ = 0.69, **(E)** *R*^2^ = 0.99, **(F)** *R*^2^ = 0.58, **(G)** *R*^2^ = 0.80, **(H)** *R*^2^ = 0.54. Values are means ± SE (*n* = 4–8).

To investigate how leaf morphological and physiological traits were associated with the levels of GAs and CKs in leaves, relationships among A_max_, N_area_, TNC, and the levels of GAs and CKs in each leaf were analyzed. There were strong positive correlations between A_max_ and N_area_ under both low and high light conditions (Figures 8A,B). N_area_ was correlated with the levels of CKs under both light conditions when the values of LowL were excluded in high light conditions (Figures 8C,D). The levels of GAs were only weakly correlated with N_area_ (Figures S6A,B).

We also tested the hypothesis that endogenous GAs affect structural LMA and TNC in the leaves. When the values of LowL were excluded, there were strong negative correlations between TNC and the levels of GAs in both light conditions (Figures 8E,F). The levels of CKs were weakly correlated with TNC (Figures S6C,D). Despite the fact that no obvious relationships were found between structural LMA and TNC in the untreated seedlings under both light conditions, a strong correlation was found between them among the treated seedlings under high light conditions (Figures 8G,H). We also analyzed relationships between the levels of GAs and CKs. While a positive correlation was found under low light conditions, consistent trends were not observed under high light conditions (Figures S6E,F).

### Changes in the C/N balance at the whole-plant level

Changes in N_mass_, C/N ratio, and TNC in leaves, stems, and roots in response to light intensity, nitrogen availability and the treatments were analyzed, and strong correlations were found among them (Figure S7). N_mass_ in leaves was highly correlated with that of stems and roots under both low light (Figure S7A) and high light conditions (Figure S7B). The C/N ratio in leaves was also highly correlated with that of stems and roots under both low light (Figure S7C) and high light conditions (Figure S7D). Although TNC in leaves was highly correlated with TNC in stems and roots under high light conditions (Figure S7F), such relationships were not found under low light conditions because TNC in leaves, stems, and roots changed only weakly in response to nitrogen availability (Figure S7E).

## Discussion

### Changes in morphological and physiological traits correspond to changes in endogenous GAs and CKS at the whole-plant level

The untreated seedlings showed typical morphological and physiological differences in response to light intensity and nitrogen availability (Figures 2–4); the stepwise increase or decrease in *L*/*R*, N_area_, PNC, A_max_, and TNC in response to nitrogen availability indicated that the experimental design of the present study was appropriate to evaluate various changes in response to nitrogen availability. The decrease in TNC in each organ in response to nitrogen availability is also a typical response to the increase in nitrogen availability (Figure 4; Stitt and Krapp, 1999).

Our previous studies showed the levels of GAs and CKs were higher under higher nitrogen availability at the whole-shoot level (Sugiura et al., 2015a). In the present study, we also observed a gradual increase in the levels of GAs and CKs with increasing nitrogen availability in the leaves of the untreated seedlings (Figures S3E–H). On the other hand, in the shoot apex, the levels of GAs gradually increased with nitrogen availability in the untreated seedlings whereas those of CKs did not (Figures S3A–D). Since the number of leaves increased with nitrogen availability (Figures S1C,D), it is possible that GAs play a more significant role in regulating the production of new leaves than CKs in the shoot apex.

In the present study, HH seedlings were subjected to three treatments to evaluate causal relationships among changes in biomass allocation, nitrogen availability, and light intensity and the levels of GAs and CKs in response to environmental variables. In addition, it was notable that Δ*L*/Δ*R* was introduced to evaluate the changes in biomass allocation between leaves and roots after the treatments. The decreased *L*/*R*, N_area_, and A_max_ and increased TNC in LowN were typical of seedlings grown under low nitrogen availability, and the increased *L*/*R* and decreased LMA and TNC in LowL were typical of seedlings grown under low light conditions, respectively (Figures 2–4). Reduced *L*/*R* by the defoliation treatment was almost recovered after 79 days by the increase and decrease in biomass allocation to leaves and roots, respectively (Figure 2). This reflected the large increase in Δ*L*/Δ*R*, an indicator of changes in biomass allocation between leaves and roots after the treatments in Def (Figure 5). The increase in N_area_ (Figure 2) and A_max_ (Figure 3) after the partial defoliation treatment was previously observed in various herbaceous and woody plants (Von Caemmerer and Farquhar, 1984; Reich et al., 1993; Turnbull et al., 2007). This study suggests that an increase in A_max_ would contribute to the recovery of leaf biomass in *P*. *cuspidatum*.

The observed decrease in the levels of GAs and CKs in LowN was reasonable since they occurred alongside the decrease in nitrogen availability in the untreated seedlings (Figures S3E,F). A remarkable finding was that the levels of GAs in both the shoot apex and leaves increased in Def. Furthermore, the levels of GAs in both the shoot apex and leaves were highly correlated with Δ*L*/Δ*R* (Figures 5A,B,E,F), while the levels of CKs in the shoot apex and leaves were less correlated with Δ*L*/Δ*R* among all of the seedlings (Figures 5C,D,G,H). These results suggest that GAs rather than CKs play a more prominent role in the regulation of biomass allocation in response to the drastic changes in environmental variables. GAs in the shoot apex seem to regulate its activity and, accordingly, the number of leaves. Similar findings have been reported by (Banyai et al., 2011), who showed that application of GA_3_ increases the number of leaves in *Artemisia annua*, while decreased levels of endogenous GAs were found to decrease the number of leaves in *S. lycopersicum* (Nagel et al., 2001b). The higher levels of GAs in the shoot apex in Def and, in spite of relatively low Δ*L*/Δ*R*, in LowL could explain the significant increase in leaf number after these treatments (Figures 5A,B and Figure S1D). These results support our hypothesis that the levels of GAs and CKs change in response to environmental variables, which cause changes in biomass allocation at the whole-plant level. Furthermore, our data suggest that GAs produced in the leaves increase *L*/*R* by promoting biomass allocation to the above-ground parts whereas CKs synthesized in the roots increase *L*/*R* by suppressing root growth. This accords with previous studies that transgenic tobacco with significantly decreased CK levels from overexpressing cytokinin oxidase in roots showed greater biomass allocation to roots (Werner et al., 2010). Moreover, other transgenic tobacco overexpressing GA20-oxidase that causes a significant increase in the activated form of GAs, GA_1_, showed greater biomass allocation to above-ground parts (Biemelt et al., 2004).

### Regulatory mechanisms of biomass allocation by GAs from the analysis of various traits and phytohormones

Analysis of the endogenous levels of phytohormones in each leaf showed age-dependent changes (Figure 6 and Figure S4). Since the levels of tZs are known to reflect the transpiration rate (Boonman et al., 2007, 2009; Reeves et al., 2007), higher levels of tZs in younger leaves may be due to the higher transpiration rate in HL and Def (Figure S4C). LowL had the lowest levels of tZs in all leaves, which reflected its low transpiration rate caused by the decrease in light intensity. The constant levels of iPs (Figure S4D), which are synthesized in the leaves, were also consistent with a previous study (Boonman et al., 2007). The contribution of iPs to the promotion of the above-ground growth in response to changes in environmental variables seems to be insignificant, since they are apparently less active than tZs in aerial organs (Kiba et al., 2013).

The strong correlations between the levels of GAs in the shoot apex and that in the youngest leaves (Figures S5A,B) suggest that GAs can be transported from the youngest leaves to the shoot apex. Many previous studies using *A. thaliana, Lolium temulentum*, and tobacco plants revealed that GAs are synthesized in leaves and transported as precursors from leaves through phloem to stems. GAs not only promote xylogenesis and fiber formation in stems (Ragni et al., 2011), but also stimulate flowering after moving to the shoot apex (King et al., 2001; Eriksson et al., 2006). Furthermore, it was also demonstrated that leaf-derived GAs move both acropetally and basipetally in stems (Dayan et al., 2012). A recent study showed that GA_12_, a biologically inactive precursor of GAs, is the major mobile signal of GAs and is activated at the site of action in *A. thaliana* (Regnault et al., 2015). In the present study, we found that the majority of GA precursors were not GA_12_ but GA_53_ in both the shoot apex and the youngest leaves (Tables 1, 2). However, both GA_12_ and GA_53_ are C-20 gibberellins and their chemical structures are almost the same. Thus, it is probable that the biosynthesis of GA_53_ is controlled in response to changes in environmental variables and GA_53_ is transported from the younger leaves through the phloem to the shoot apex as a major mobile signal in *P. cuspidatum*.

### Regulation of morphological and physiological traits by GAs and CKS at each leaf level

We also found age-dependent changes in the morphological and physiological traits in each leaf. The strong correlations between A_max_ and N_area_ across the untreated and treated seedlings (Figures 8A,B) suggest that the changes in A_max_ observed were mainly dependent on the changes in N_area_ and that photosynthetic rate can be regulated plastically through the regulation of N_area_ in each leaf. The further correlation between N_area_ and the levels of CKs (Figures 8C,D) suggests that N_area_ in each leaf is optimized through changes in the levels of CKs responding to changes in environmental variables. This is because two of the major functions of CKs are the retardation of leaf senescence (Gan and Amasino, 1995) and the regulation of nitrogen remobilization by decreasing protein degradation (Criado et al., 2009). From these results, it was shown that the level of CKs in leaves can be an indicator of leaf photosynthetic capacity under the same light conditions.

It was reported that endogenous GAs regulate leaf growth by promoting cell division in maize (Nelissen et al., 2012), and exogenous application of GA_3_ also promotes leaf sheath growth in concurrence with starch consumption in rice (Matsukura et al., 1998). Thus, it is suggested that promotion or suppression of above-ground growth by GAs also causes consumption or accumulation of TNC in leaves as shown in the negative correlation between TNC and the levels of GAs (Figures 8E,F). The positive correlations between structural LMA and TNC in the leaves of treated seedlings (Figures 8G,H) were consistent with our previous study, in which we conducted reciprocal grafting experiments using two varieties of *Raphanus sativus* with different sink activities (Sugiura et al., 2015b). We considered the possibility that excess TNC caused by changes in sink activity can be converted into structural components such as the cell wall. In the present study, it is also probable that rapid changes in the sink–source balance caused by defoliation and low nitrogen treatments could cause both the accumulation of TNC and the conversion of TNC into structural components. On the other hand, there was no positive correlation between structural LMA and TNC among the untreated seedlings under both light conditions (Figures 8G,H). Thus, the conversion of excess TNC into structural components may not occur in untreated seedlings which maintain a constant sink–source balance even in HL seedlings with the highest TNC.

### Regulatory mechanism of C/N ratio and TNC at the whole-plant level

Concomitant changes in N_mass_, C/N ratio, and TNC in each organ among the untreated and treated seedlings are also interesting (Figure S7). For example, when N_mass_ in the leaves was changed by the treatments, N_mass_ in stems and roots are also changed accordingly. Since GAs may be transported from the leaves (Figures S5A,B) and involved in the consumption of TNC (Figures 8E,F), it is also possible that GAs transported from the leaves regulate the observed concomitant changes in the traits of stems and roots. Therefore, future work should clarify whether endogenous levels of GAs in leaves, stems, and roots were regulated in a coordinated manner.

## Conclusion

The present study revealed the causal relationships between the endogenous levels of GAs and CKs and biomass allocation that change plastically in response to changes in environmental variables. Furthermore, the present data suggest that GAs are transported from the leaves to the shoot apex to regulate biomass allocation. It is also suggested that LMA and TNC of the leaves and the photosynthetic rates in each leaf were partly regulated by the endogenous levels of GAs and CKs, respectively. These results fully support our hypothesis that GAs and CKs are key regulatory factors that control biomass allocation and morphological and physiological traits of leaves in response to changes in environmental variables.

## Author contributions

DS designed the experiments, DS, MK, and HS performed the experiments, and DS wrote the paper.

### Conflict of interest statement

The authors declare that the research was conducted in the absence of any commercial or financial relationships that could be construed as a potential conflict of interest.
