# Supplementary material for: Phytohormonal Regulation of Biomass Allocation and Morphological and Physiological Traits of Leaves in Response to Environmental Changes in Polygonum cuspidatum
Source: Front Plant Sci. 2016 Aug 9;7:1189. doi: 10.3389/fpls.2016.01189 (PMC4977362; doi:10.3389/fpls.2016.01189)
Supplement: Supplementary file 1 [file DataSheet1.DOCX]

Supplementary Material

Phytohormonal regulation of biomass allocation and morphological and physiological traits leaves in response to environmental changes in *Polygonum cuspidatum*

Daisuke Sugiura*, Mikiko Kojima, Hitoshi Sakakibara

*** Correspondence:** Corresponding Author: daisuke.sugiura@gmail.com

**FIGURE. S1.** Biomass of leaves, stems, and roots **(A, B)** and number of leaves **(C, D)** in *Polygonum cuspidatum*. See text for abbreviations. Bars from top to bottom represent values of leaves (white bar), stems (grey bar), and roots (black bar) in **(A, B)**. The white bar inside the value for the number of leaves in Def **(D)** represents the value just after defoliation. Values are means + SE (*n*=4–8). Letters indicate signiﬁcant differences among nitrogen treatments in low light (LL, LM, and LH) and high light (HL, HM, and HH) (one-way ANOVA, *P*<0.05, followed by Tukey’s test, *P*<0.05). Asterisks indicate signiﬁcant differences in Def, LowN, and LowL compared with HH (Student’s *t* test, **P*<0.05; ***P*<0.01).

**FIGURE. S2.** Relationships between endogenous levels of GA_1_ and GA precursors in the shoot apex and those in all leaves of LL, LM, LH, HL, and HM and the youngest leaves of HH, Def, LowN and LowL in **(A, C)** low light and **(B, D)** high light conditions in *Polygonum cuspidatum*. Sum of the levels of GA_12_, GA_24_, GA_9_, GA_53_, GA_44_, GA_19_, and GA_20_ is defined as GA precursors. See text for abbreviations. Closed rectangles, triangles, and circles denote LL, LM, and LH, and open rectangles, triangles, and circles denote HL, HM, and HH, respectively. Grey triangles, grey rectangles, and grey diamonds denote Def, LowN, and LowL, respectively. Solid lines represent regression lines; **(A)** *R*^2^=0.87, **(B)** *R*^2^=0.74, **(C)** *R*^2^=0.99, **(D)** *R*^2^=0.68. Values obtained from the all leaves are presented for LL, LM, LH, HL, and HM, and those from the youngest leaves are presented for HH, Def, LowN, and LowL **(B, D)**. Values are means ± SE (*n*=4–8).

**FIGURE. S3.** Endogenous levels of GAs and CKs in the shoot apex **(A, B, C, D)** and leaves **(E, F, G, H)** in *Polygonum cuspidatum*. See text for abbreviations. For GAs and CKs in leaves, values obtained from the all leaves are presented for LL, LM, LH, HL, and HM, and weighted mean values of all the leaves are presented for HH, Def, LowN, and LowL. Values are means + SE (*n*=4–8). Letters indicate signiﬁcant differences among nitrogen treatments in low light (LL, LM, and LH) and high light (HL, HM, and HH) (one-way ANOVA, *P*<0.05, followed by Tukey’s test, *P*<0.05). Asterisks indicate signiﬁcant differences in Def, LowN, and LowL compared with HH (Student’s *t* test, **P*<0.05; ***P*<0.01). Values are means + SE (*n*=4–8).

**FIGURE. S4.** Leaf nitrate content **(A)**, leaf nitrogen content per mass **(B)** and endogenous levels of trans-zeatin type CKs (tZs) **(C)**, N6-(D2-isopentenyl) adenine type CKs (iPs) **(D)**, indole-3-acetic acid (IAA) **(E)**, abscisic acid (ABA) **(F)**, salicylic acid (SA) **(G)**, and jasmonic acid (JA) **(H)** in each leaf of *Polygonum cuspidatum*. See text for abbreviations. Open circles, grey triangles, grey rectangles, and grey diamonds denote HH, Def, LowN, and LowL, respectively. Numbers on the *x*-axes denote the leaf position (see text for details). Values are means ± SE (*n*=4–8).

**FIGURE. S5.** Relationships between endogenous levels of GAs and CKs in the shoot apex and those in all leaves of LL, LM, LH, HL, and HM and the youngest leaves of HH, Def, LowN and LowL in **(A, C)** low light and **(B, D)** high light conditions in *Polygonum cuspidatum*. See text for abbreviations. Closed rectangles, triangles, and circles denote LL, LM, and LH, and open rectangles, triangles, and circles denote HL, HM, and HH, respectively. Grey triangles, grey rectangles, and grey diamonds denote Def, LowN, and LowL, respectively. Solid lines represent regression lines; **(A)** *R*^2^=0.99, **(B)** *R*^2^=0.58, **(C)** *R*^2^=0.62, **(D)** *R*^2^=0.52. Values obtained from the all leaves are presented for LL, LM, LH, HL, and HM, and those from the youngest leaves are presented for HH, Def, LowN, and LowL **(B, D)**. Values are means ± SE (*n*=4–8).

**FIGURE. S6** Relationships between endogenous levels of GAs in leaves and N_area_, those between total non-structural carbohydrates (TNC) and endogenous levels of CKs, and those between endogenous levels of CKs in leaves and those of GAs in leaves in low light **(A, C, E)** and high light conditions **(B, D, F)** in *Polygonum cuspidatum*. See text for abbreviations. Values obtained from the all leaves are presented for LL, LM, LH, HL, and HM, and those from each leaf are presented for HH, Def, LowN, and LowL. Closed rectangles, triangles, and circles denote LL, LM, and LH, and open rectangles, triangles, and circles denote HL, HM, and HH, respectively. Grey triangles, grey rectangles, and grey diamonds denote Def, LowN, and LowL, respectively. Solid curves represent regression curves for LL, LM, and LH **(A, C, E)** and HL, HM, HH, Def, and LowN **(B, D, F)** where the values of LowL were excluded in **(D, F)**; **(A)** *R*^2^=0.98, **(B)** *R*^2^=0.17, **(C)** *R*^2^=0.98, **(D)** *R*^2^=0.65, **(E)** *R*^2^=0.99, **(F)** *R*^2^=0.57. Values are means ± SE (*n*=4–8).

**FIGURE. S7.** Relationships between nitrogen content per mass (N_mass_) **(A, B)**, those between C/N ratio **(C, D)**, and those between non-structural carbohydrates (TNC) **(E, F)** in leaves, stems, and roots. See text for abbreviations. Values obtained from the all leaves are presented for LL, LM, LH, HL, and HM, and weighted mean values of all the leaves are presented for HH, Def, LowN, and LowL. Closed rectangles, triangles, and circles denote LL, LM, and LH, and open rectangles, triangles, and circles denote HL, HM, and HH, respectively. Grey triangles, grey rectangles, and grey diamonds denote Def, LowN, and LowL, respectively. Solid, dashed, and dot-dash lines represent relationships between the values of roots and leaves, those between the values of stems and leaves, and those between the values of stems and roots, respectively; **(A)** *R*^2^=0.98, 0.96, 1.00, **(B)** *R*^2^=0.94, 0.95, 0.99, **(C)** *R*^2^=0.99, 0.95, 0.98, **(D)** *R*^2^=0.99, 0.99, 1.00, **(E)** *R*^2^=0.77, 0.69, 0.99, **(F)** *R*^2^=0.85, 0.90, 0.98. Values are means ± SE (*n*=4–8).
